# Pentoxifylline reduces the inflammatory process in diabetic rats: relationship with decreases of pro-inflammatory cytokines and inducible nitric oxide synthase

**DOI:** 10.1186/s12950-015-0080-5

**Published:** 2015-04-23

**Authors:** Francisca Adilfa de Oliveira Garcia, Jéssica Farias Rebouças, Teresa Queiroz Balbino, Teresinha Gonçalves da Silva, Carlson Hélder Reis de Carvalho-Júnior, Gilberto Santos Cerqueira, Gerly Anne de Castro Brito, Glauce Socorro de Barros Viana

**Affiliations:** Faculty of Medicine Estácio of Juazeiro do Norte, Juazeiro do Norte, Brazil; Federal University of Pernambuco, Recife, Brazil; Faculty of Medicine of the Federal University of Ceará, Rua Barbosa de Freitas, 130/1100, Fortaleza, CEP: 60170-020 Brazil

**Keywords:** Diabetes mellitus, Pentoxifylline, Oxidative stress, Inflammation, Cytokines

## Abstract

Studies suggest that inflammation is a key factor in the pathogenesis of diabetes mellitus. Pro-inflammatory cytokines, such as IL-6 and TNF-alpha, are produced by adipose tissue in large quantities, in obese and especially in diabetic individuals. Pentoxifylline (PTX) is a non-selective phosphodiesterase inhibitor with anti-inflammatory and antioxidant actions that may contribute to alleviate diabetes side effects, as neuropathy, retinopathy and nephropathy. This study aims to investigate PTX anti-inflammatory effects on the carrageenan-induced paw edema model, in alloxan-induced diabetic rats. Diabetic animals (male Wistar rats, 200–250 g) were daily treated with PTX (25, 50, 100 mg/kg, p.o.), glibenclamide (GLI, 5 mg/kg, p.o., as reference) or water, for 5 days. Afterwards, carrageenan-treated paws were dissected, their skin removed and the tissue used for preparation of homogenates and measurements of IL-6 and TNF-alpha by Elisa. Serum levels of nitrite were also determined and paw slices used for iNOS immunohistochemistry assays. We showed that diabetic rats presented an amplification of the inflammatory response, as related to non-diabetic rats, what was evident 48 h after the edema-induction. The PTX-treatment of diabetic rats reduced glycemia (as related to untreated-diabetic ones) and the paw edema. It also brought edema volumes to values similar to those of non-diabetic rats, at the same observation time. The increased TNF-alpha and IL-6 levels in paws of untreated-diabetic rats were reduced in diabetic animals after PTX treatments. Besides, the increased levels of nitrite in the serum of diabetic rats were also decreased by PTX. Furthermore, a higher number of iNOS immunostained cells was demonstrated in paw tissues from untreated-diabetic rats, as related to those of PTX-treated diabetic animals. Our results show that PTX reduces inflammatory parameters, as pro-inflammatory cytokines and iNOS expression, indicating the potential benefit of the drug for the treatment of diabetes and related pathologic conditions.

## Introduction

There is increasing evidence that diabetes is associated with an enhanced inflammatory state and that inflammatory cells contribute to atherosclerotic lesion initiation and disruption. Furthermore, data support the important role of inflammation in atherosclerosis associated with type 1 and type 2 diabetes and insulin resistance, as well. Thus, Navarro and Mora, 2005 [1] showed that inflammation and more specifically pro-inflammatory cytokines play a determinant role in the development of microvascular diabetic complications. For instance, diabetic neuropathy develops as the result of hyperglycemia-induced metabolic, enzymatic and microvascular changes.

Pro-inflammatory cytokines are produced locally by resident and infiltrating cells, and these molecules exhibit pleiotropic effects on homeostasis of glia and neurons, in the central, peripheral and autonomic nervous systems. In addition, changes induced by chronic hyperglycemia lead to dysregulation of these cytokines. Experimental investigations have demonstrated that mRNA expression for TNF-alpha increases significantly in kidneys from diabetic rats, as related to those from normal animals [1,2]. This cytokine is cytotoxic to glomerular, mesangial and epithelial cells and may induce significant renal damage [3].

Furthermore, clinical and experimental studies have shown that hyperglycemia results in advanced glycation end-products (AGE) accumulation in tissues from diabetic patients, binding to the cellular receptor RAGE. This AGE/RAGE interaction initiates a signaling cascade, involving an increase of the nuclear transcription factor (NF-κB). Consequently, an additional increase in oxidative stress and production of pro-inflammatory cytokines occur [4]. In the beginning, the inflammatory reaction results in increased levels of TNF-alpha, IL-1beta and IL-6 that interact with acute phase proteins [5]. With the disease development, the persistence of increased abnormal levels of these proteins leads to a state of mild chronic inflammation what could be a factor responsible for the accelerated atherosclerosis of the diabetic population [6]. Thus, a subclinical inflammation precedes diabetes and, for that, pro-inflammatory cytokines play an important role.

Moreover, intervention studies confirm the role of inflammation in the pathogenesis of diabetes and vascular complications, and their association to inflammatory processes opens new clinical perspectives for diabetes diagnosis and treatment. Commonly, type 1 and type 2 diabetes are considered inflammation-associated diseases, as there is an increase in pro-inflammatory cytokines in the blood of affected patients [1,7,8]. Diabetic individuals show elevated plasma concentrations of pro-inflammatory cytokines and serum amyloid acute phase protein A [9]. These cytokines and proteins are produced by adipose tissues, in part originated from leukocytes and T cells. Such patients show a constant subclinical inflammatory state that is directly correlated to chronic complications of the disease [8,10-12].

In the 90s, a number of lines of research raised the concept that diabetes was associated to the activation of the immune system and subsequent inflammation. One of those related obesity to the secretion of pro-inflammatory cytokines and proteins, as TNF-alpha, IL-6, leptin and adiponectin. Others indicated that pro-inflammatory cytokines, as IL-6 and TNF-alpha, affected the secretion and efficiency of insulin [13-17].

Pentoxifylline (PTX) is a nonselective phosphodiesterase inhibitor and a methylxanthine derivative known for its anti-inflammatory and immunomodulator effects [18]. This drug is able to inhibit TNF-alpha production in macrophage, monocytes and T lymphocytes, *in vitro* and *in vivo* [19-21]. In some studies, the use of PTX is based on its capacity for inhibiting pro-inflammatory cytokines production, present in diabetes since the beginning of the disease [22]. However, this is not a matter of consensus, since it was demonstrated [23] that, despite lowering TNF-alpha levels, PTX did not improve the vascular function in either conduit or resistance vessels, in a group of type II diabetic subjects. Others [24] also observed that PTX did not improve the ocular blood flow in healthy subjects.

On the other hand, PTX treatments were shown to confer neurovascular benefits, in experimental diabetic neuropathy linked, at least partly, to cyclooxygenase-mediated metabolism [25]. Previously [26], a group of 10 diabetic atherosclerotic patients demonstrated a significant increase in exercise tolerance, after PTX treatment, and 8 of them also presented a significant increase in arterial blood flow. Others [27] showed that PTX treatment of diabetic patients increased retinal capillary blood flow velocity. These alterations in blood flow could also contribute to edema volume and inflammation. Furthermore, increasing evidences [28] show that oxidative stress is associated with the pathogenesis of several diseases including diabetes and others inflammation-related diseases contributing to the concept that oxidative stress is the final common pathway by which risk factors exert their deleterious effects.

In the present study, PTX effects were compared to those of glibenclamide (GLI), a sulfonylurea largely used in clinics to T2 diabetes. It is a second generation sulfonylurea that lowers blood glucose by increasing insulin secretion from pancreatic beta cells. It also has other extra-pancreatic hypoglycemic actions that are important in case of prolonged therapy.

The objectives of the present work were to evaluate PTX effects on the inflammatory response of diabetic rats, in the model of alloxan-induced diabetes as related to that of GLI used as a reference drug, attempting to related this effect to the reduction of pro-inflammatory cytokines induced by the drug. Thus, measurements of IL-6 and TNF-alpha, in paws and sera from untreated-diabetic or diabetic rats after PTX or GLI treatments were performed. Furthermore, serum levels of nitrite and IL-6, as well as, immunohistochemistry assays for iNOS in diabetic rat paws were also carried out.

## Methods

### Drugs and reagents

Pentoxifylline (Trental) was purchased from Sanofi-Aventis Laboratory, São Paulo, Brazil. Glibenclamide was from EMS S/A Laboratory, São Paulo, Brazil. Alloxan and carrageenan type IV were from Sigma-Aldrich (Saint Louis, MO, USA). Cytokine kits were from eBioscience (San Diego, CA, USA), and BD Bioscience (São Paulo, Brazil) for TNF-alpha and IL-6, respectively. All other drugs and reagents were of analytical grade.

### Animals

Male Wistar rats (200–250 g) from the Animal House of the Faculty of Medicine Estácio of Juazeiro do Norte were maintained under a 12 h/12 h light/dark cycle and with food and water *ad libitum*. Experiments were approved by the Ethical Committee on Animals Research, of the Faculty of Medicine of the Federal University of Ceará, under the number 01⁄2011 and performed according to ethical principles established in the Guide for the Care and Use of Laboratory Animals, USA, 1986.

### Carrageenan-induced paw edema in diabetic rats

Carrageenan-induced inflammation in the rat paw is a classical model of edema formation and hyperalgesia, largely used in studies of drugs with anti-inflammatory activity [29]. Diabetes was induced by an alloxan injection (40 mg/kg) into the penial vein. After 48 h, blood was collected for glucose measurements and only animals with glycemia equal or higher than 250 mg/dL were used. The animals were divided into the following groups: untreated-diabetic and diabetic treated with PTX (25, 50, 100 mg/kg, p.o.) or glibenclamide (GLI, 5 mg/kg, p.o., as reference). Whenever needed, a normal control group (non-diabetic animals) was included for comparison. Treatments started 48 h after the alloxan injection and continued for 5 days, when the animals were subjected to carrageen-induced paw edema, 1 h after the drug last administration. The edema was induced by the injection of 40 μL 1% carrageenan solution into the animal’s right hind paw. Measurements of the paw volume were done by means of a plethysmometer (Ugo Basile, Italy), immediately prior to the carrageenan injection (zero time) and at 1, 2, 3, 4 and 48 h after. The paw edema volume was determined by the difference between the final and initial volumes.

### Cytokine measurements (TNF-alpha and IL-6) in paws and sera from diabetic rats

After edema measurements, the animals were euthanized and sections from their carrageenan-treated paws or sera were homogenized in PBS solution and centrifuged (7000 rpm, 5 min). The supernatants were used for cytokine determinations, following the manufacturers’ instructions. Briefly, 48 μL primary antibody (anti-TNF-alpha or anti-IL-6 fom DBioscience) were diluted in 12 mL coating buffer solution. Then, the mixture 100 μL were plated in 96-well plates and, after overnight incubation at 4°C, the primary antibody was removed by aspiration and the wells washed with PBS/Tween-20 solution. After washes, the assay diluent 200 μL/well were added and the mixture left at RT for 1 h. After new washes, 100 μL secondary antibody (48 μL stock solution in 12 mL assay diluent) were added and left standing, at RT for 1 h. The washes were also carried out 1 h later, followed by the addition of 100 μL avidin-HRP solution (prepared as 48 μL avidin-HRP in 12 mL assay diluent). The mixture was left standing at RT for 30 min, followed by new washes and the addition of 100 μL substrate solution. After 15 min, a 10% H_2_SO_4_ solution (50 μL) was added to the mixture, and the readings performed at 450 nm in a microplate reader. The standard curve was carried out in a microplate reader with initial concentrations of 2000 pg/mL for TNF-alpha and 5000 pg/mL for IL-6.

### PTX effects on nitrite contents in diabetic rat serum

Nitrite determinations were carried out in sera from diabetic rats, before and after PTX treatments for 5 days and, for that, the Griess method was used. Briefly, 50 μL samples (supernatants from blood after centrifugation at 2000 rpm, 10 min) were plated in 96-well plates. The standard curve was performed with a nitrite solution, at several different concentrations (prepared from a 100 μM stock solution). Fifty microliters Griess reagent were added to the samples and the mixture was incubated for 10 min under light protection. After this time, measurements were carried out in a microplate reader at 540 nm. The results were expressed as pg/mL.

### Immunohistochemistry assays for iNOS in diabetic rat paws

For these, the streptavidin-biotin-peroxidase method was used. Five days after PTX treatments of diabetic animals and 1 h after its last administration, the animals were euthanized and sections (5 μm) of the carrageenan-injected hind paw were immersed in 10% formalin for 24 h and inserted into paraffin blocks. The sections were then deparaffinized, dehydrated in xylol and ethanol, and immersed in 0.1 M citrate buffer (pH 6) under microwave heating for 18 min, for antigen recovery. After cooling at RT for 20 min, the sections were washed in PBS, followed by a 15 min blockade of the endogenous peroxidase with a 3% H_2_O_2_ solution. The sections were incubated overnight at 4°C with rabbit primary antibodies (anti-iNOS, 1:200 dilutions) in PBS-BSA. At the next day, the sections were washed in PBS and incubated for 30 min with the secondary biotinylated rabbit antibody (anti-IgG, 1:200 dilution) in PBS-BSA. After washing in PBS, the sections were incubated for 30 min with the conjugated streptavidin peroxidase complex (ABC Vectastain® complex, Vector Laboratories, Burlingame, CA, USA). After another washing with PBS, the sections were stained with 3,3′diaminobenzidine-peroxide (DAB) chromophore, counter-stained with Mayer hematoxylin, dehydrated and mounted in microscope slides for analyses.

### Statistical analyses

All results are presented as means ± SD. One-way ANOVA followed by Tukey’s as the *post hoc* test were used, for multiple comparisons, and the two-tailed paired Student’s *t* test for comparison between two means. To determine if there is a significant difference between two means with equal sample sizes, the Tukey’s method uses a formula which calculates the *q* value by taking the difference between two sample means and dividing it by the standard error where *q* represents the “Studentized” range value. The data were considered significant at p < 0.05.

## Results

### Evaluation of PTX effects on glycemia in alloxan-induced diabetic rats

**Figure 1:**
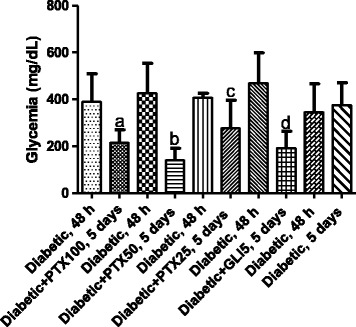
Pentoxifylline (PTX, 25, 50 and 100 mg/kg) significantly decreases glycemia in diabetic rats, after 5-day treatments. Each pair of bars represents the same diabetic group 48 h after alloxan-induced diabetes, without (untreated-diabetic rats) or with 5-day treatments with PTX or glibenclamide (GLI, 5 mg/kg). The values are means ± SD from 5 to 15 animals. a. p < 0.0002; b. p < 0.0001; c. p < 0.0313. All vs. untreated-diabetic rats (paired, two-tailed Student’s *t* test).

These experiments were carried out in untreated-diabetic rats and diabetic animals treated with PTX (25, 50 and 100 mg/kg, p.o.) or GLI (5 mg/kg, p.o., as reference) for five days (5 to 15 animals per group). Because of the short treatment-time, decreases in glycemia ranged from 32 to 67% after PTX treatments and around 59% for the GLI5 group, as related to each group before treatments (Figure 1).

### Anti-inflammatory effects of PTX on the carrageenan-induced edema in diabetic rats

**Figure 2:**
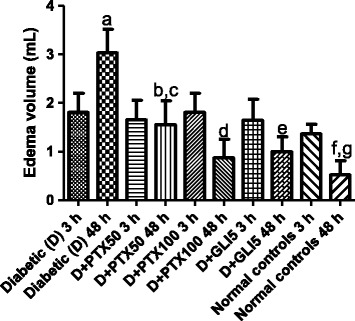
Pentoxifylline (PTX, 50 and 100 mg/kg) treatments for 5 days significantly decrease edema volumes in diabetic rats, 48 h after the carrageenan injection. Glibenclamide (GLI, 5 mg/kg) and normal controls (non-diabetic rats) were included for comparisons. The values are means ± SD from 8 to 13 animals. a. vs. diabetic 3 h, q = 9.519; b. vs. diabetic 48 h, q = 11.49; c. vs. diabetic + PTX100, 48 h, q = 5.659; d. vs. diabetic, 48 h, q = 16.47; e. vs. diabetic 48 h, q = 12.90; f. vs. diabetic 48 h, q = 17.54; g. vs. normal 48 h, q = 6.049 (One-way ANOVA ans Tukey’s as the *post hoc* test).

Increases of 1.3- and 5.7-fold in edema volumes of untreated-diabetic rats were observed, at 3 and 48 h after the carrageenan injection, as related to normal controls (non-diabetic animals). Similar increases in edema volumes were observed 3 h later in diabetic groups, after PTX (50 and 100 mg/kg) or GLI (5 mg/kg) treatments, as related to untreated-diabetic groups at the same period. However, reductions of edema volumes, ranging from 50 to 65% were demonstrated in diabetic groups, after PTX50, PTX100 or GLI5 treatments, as related to the untreated-diabetic group, 48 h after the carrageenan administration. The edema volumes in treated-diabetic groups, at this period, were still higher (1.9- to 2.9-fold) when related to normal controls. These data suggest, as expected, a longer edema duration in diabetic rats, as compared to non-diabetic animals and, although treatments with PTX or GLI change this pattern towards normality, the edema was not completely dismissed (Figure 2).

### Effects of PTX on TNF-alpha and IL-6 concentrations in paws and sera from diabetic rats after carrageenan-induced edema

**Figure 3:**
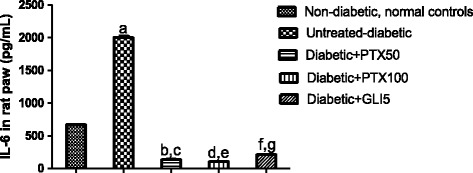
Pentoxifylline (PTX, 50 and 100 mg/kg) treatments of diabetic rats for 5 days significantly decrease IL-6 levels in rat paws, at both doses, as related to untreated-diabetic animals and to normal controls (non-diabetic animals). Glibenclamide (GLI, 5 mg/kg) was included for comparisons. Measurements were done in homogenates and the values are means ± SD from groups of three animals each. a. vs. normal controls, q = 233.9; b. vs. normal controls, q = 98.47; c. vs. untreated-diabetic group, q = 332.4; d. vs. normal controls, q = 92.99; e. vs. untreated-diabetic group, q = 326.9; f. vs. normal controls, q = 80.20; g. vs. untreated-diabetic group, q = 314.1 (One-way ANOVA followed by Tukey’s as the *post hoc* test).

**Figure 4:**
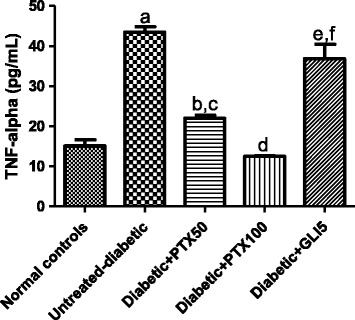
Pentoxifylline (PTX, 50 and 100 mg/kg) treatments of diabetic rats for 5 days significantly decrease TNF-alpha levels in rat paws, at both doses, as related to untreated-diabetic animals and to normal controls (non-diabetic rats). Measurements were done in homogenates and the values are means ± SD from groups of three animals each. Glibenclamide (GLI, 5 mg/kg) was used as reference. a. vs. normal controls, q = 26.65; b. vs. normal controls, q = 6.476; c. vs. untreated-diabetic group, q = 20.18; d. vs. untreated-diabetic group, q = 29.09; e. vs. normal controls, q = 20.46; f. vs. untreated-diabetic group, q = 6.194 (One-way ANOVA and Tukey’s as the *post hoc* test).

**Figure 5:**
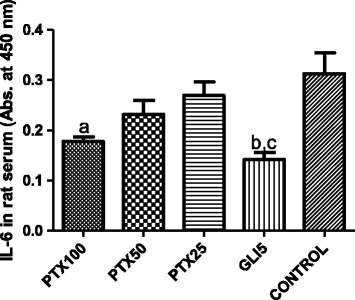
Pentoxifylline (PTX, 25, 50 and 100 mg/kg) treatments of diabetic rats for 5 days decrease serum IL-6 concentrations, as related to untreated-diabetic animals. Glibenclamide (GLI, 5 mg/kg) was included as reference. a. vs. untreated-diabetic group (control), q = 4.365; b. vs. Control, q = 5.128; c. vs. PTX25, q = 4.676 (One-way ANOVA and Tukey’s as the *post hoc* test).

Experiments were performed with homogenates from diabetic rat paws or sera, after 5-day treatments with PTX. The results showed increases of almost 3-fold in TNF-alpha concentrations in paws from untreated-diabetic rats (3 animals per group), as related to normal controls (non-diabetic animals). TNF-alpha values decreased in a dose-dependent fashion in paws from diabetic rats, after PTX treatments (49 and 71%, after 50 and 100 mg/kg, respectively), as related to the untreated-diabetic group. Surprisingly, the decrease in diabetic groups after GLI treatments was only 15% and the values were significantly higher (more than 2-fold), as related to those of normal controls. Although TNF-alpha concentrations were close to normal ones in diabetic rats treated with PTX, at the dose of 50 mg/kg, the decreases were even higher than those from normal controls, at the PTX dose of 100 mg/kg (Figure 3). A 3-fold increase was observed in IL-6 concentrations in untreated-diabetic rat paws, as related to normal controls. PTX (50 and 100 mg/kg) treatments of diabetic rats decreased IL-6 concentrations by more than 90%, as related to the untreated-diabetic group. A similar result (90% decrease) was seen after treatment of the diabetic group with GLI5 (Figure 4). Unlikely GLI that reduced by 67% IL-6 contents in sera from diabetic rats, changes were observed only after PTX treatments at the higher dose (Figure 5).

### PTX effects on nitrite concentrations in diabetic rat sera

**Figure 6:**
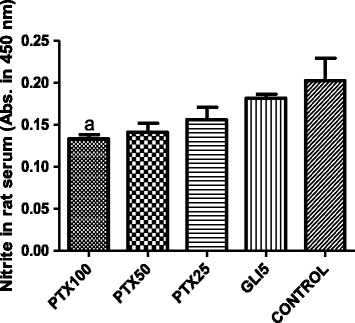
Pentoxifylline (PTX, 25, 50 and 100 mg/kg) treatments of diabetic rats for 5 days decrease serum nitrite concentrations, as related to untreated-diabetic animals. Glibenclamide (GLI, 5 mg/kg) was included as reference. The values are means ± SD from 4 to 7 animals per group. a. q = 4.749 vs. untreated-diabetic control rats (One-way ANOVA and Tukey’s as the *post hoc* test).

Decreases of 51, 57 and 65% were demonstrated in nitrite concentrations in sera from diabetic rats, after 5-day PTX treatments with the doses of 25, 50 and 100 mg/kg, respectively, as related to untreated-diabetic rats (4 to 7 animals per group). A lower decrease (37%) was observed in the diabetic group after GLI5 treatments (Figure 6).

### Immunohistochemistry for iNOS in diabetic rat paws

**Figure 7:**
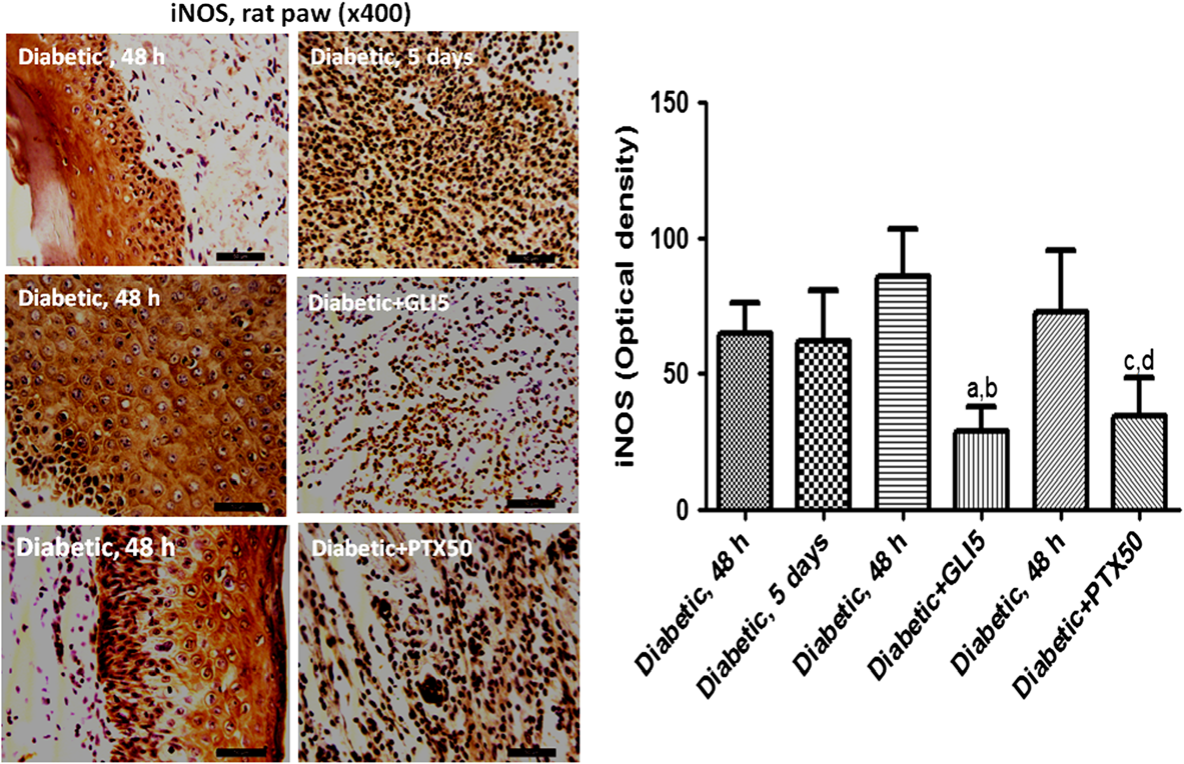
Representative photomicrographs of immunohistochemistry assays for iNOS (x400) showing that pentoxifylline (PTX, 50 mg/kg) or glibenclamide (GLI, 5 mg/kg) 5-day treatments decrease the immunoreactivity for iNOS in paws from diabetic rats, as related to those from the same group before treatments (48 h after diabetes induction). The histograms show the relative optical density (means ± SD) determined from 5–9 fields, by the Image J software. a. vs. the same diabetic group at 48 h, q = 9.106; b. vs. the untreated 5-day diabetic group, q = 5.868; c. vs. the same diabetic group at 48 h, q = 6.746; d. vs. the untreated 5-day diabetic group, q = 4.842 (One-way ANOVA and Tukey’s as the *post hoc* test).

Figure 7 shows representative photomicrographs of untreated-diabetic rats and diabetic rats treated with PTX (50 and 100 mg/kg) or glibenclamide (GLI, 5 mg/kg, as reference), for 5 days. The results presented higher immunoreactivity for iNOS in paw slices from untreated-diabetic rats. The numbers of immunopositive cells were lower in the diabetic group, mainly after the higher PTX dose, as measured by the Image J software. The GLI5 group also presented a lower immunoreactivity, as related to the same group before treatments (untreated-diabetic group).

## Discussion

Pentoxifylline, a methylxanthine derivative and phosphodiesterase inhibitor, is primarily used for the treatment of peripheral arterial insufficiency. Data derived from human studies and animal models provide a robust scientific basis for PTX as an antiproteinuric agent. Available evidences indicate that PTX may decrease proteinuria in patients with diabetic nephropathy [30]. Furthermore, PTX appears to have anti-inflammatory properties with demonstrated efficacy in decreasing serum and urinary TNF-alpha levels, in patients with diabetic nephropathy [31,32].

In the present work, we studied the effects of pentoxifylline (PTX) on the inflammatory response of diabetic rats, as evaluated by the carrageenan-induced paw edema. In this classical model of acute inflammation, the development of edema is described as a biphasic event in which various mediators operate in sequence to produce the inflammatory response [33]. The initial phase (0–1 h after the carrageenan injection) is attributed to the release of histamine, 5-hydroxytriptamine and bradykinin. In contrast, the second and accelerating phase (1–6 h after the carrageenan injection) has been correlated to the production of prostaglandins and attributed to the induction of COX-2 in the hind paw [34]. Additionally, local neutrophil infiltration contributes to the carrageenan-induced inflammatory response, by producing oxygen-derived and hydroxyl free radicals [35-38].

Another important mediator, in acute and chronic inflammation is nitric oxide (NO). NO is a potent vasodilator and its involvement in the inflammatory response may be related to the ability to increase vascular permeability and edema, through changes in the local blood flow [39,40]. Other results [36] suggest that NO is involved in the development of inflammation, at early time points following carrageenan administration, and that NO produced by iNOS is involved in the maintainance of the inflammatory response, at later time points.

Numerous studies have shown that low-grade inflammation is associated with the risk of developing type 2 diabetes [1,41,42]. Cytokines, as IL-6 and TNF-alpha, are elevated in diabetic patients, suggesting that the pattern of circulating inflammatory cytokines modifies the risk for diabetes and is correlated with insulin resistance [7,12].

Two cellular processes affected by diabetes are inflammation and apoptosis. In particular, dysregulation of TNF-alpha and the formation of advanced glycation products, both of which occur at higher levels in diabetic humans and animal models, potentiate inflammatory responses [43]. Although it is accepted that chronic subclinical inflammation is part of the insulin resistance syndrome [44], the mechanisms by which it evokes diabetes are not clear. The adipose tissue can synthesize and release pro-inflammatory cytokines, as TNF-alpha, IL-1 and IL-6, and these inflammatory markers are associated with body fat mass and involved in multiple metabolic pathways relevant to insulin resistance [45]. Evidences [46] indicate that hyperglycemia increases circulating IL-6 and TNF-alpha levels, by an oxidative mechanism, and this effect is more pronounced in subjects with impaired glucose tolerance, suggesting a causal role for hyperglycemia in the immune activation of diabetes.

Furthermore, experimental investigations have demonstrated that mRNA expression for TNF-alpha is increased in kidneys from diabetic rats, as compared with kidneys from normal rats. This cytokine is cytotoxic to glomerular, mesangial and epithelial cells and may induce significant renal damage [3]. PTX has been shown to inhibit the accumulation of TNF-alpha mRNA and the transcription of the TNF-alpha gene, suppressing the production of this cytokine. This indicates the efficacy of PTX in different models of renal diseases [47,48].

We showed that the inflammatory response, as evaluated by the acute model of carrageenan-induced edema, decreases in intensity and duration in diabetic rats, after PTX treatments. This result is probably related to reduced cytokine levels, as TNF-alpha and IL-6, induced by PTX in the diabetic rat paws, as related to paws from untreated-diabetic rats. Studies by Gonzalez et al., 2012 [49] suggest that hyperglycemia downregulates CD33 expression (membrane receptor, expressed by monocytes) and triggers the spontaneous secretion of TNF-alpha by peripheral monocytes, involving the generation of ROS and the upregulation of the suppressor of cytokine signaling protein-3. According to these authors, these data support the importance of blood glucose control for innate immune function and suggest the participation of CD33 in the inflammatory profile associated with diabetes.

Evidences [50] indicated increases in TNF-alpha and IL-6 levels, in rat paws subjected to carrageenan-induced edema, results that are similar to ours. The hypoglycemic activity of PTX, as observed in the present work, is also probably involved with the different profile of the inflammatory response of PTX-treated diabetic rats, as related to untreated diabetic rats. Although we observed decreases in TNF-alpha and IL-6 concentrations in diabetic rat paws, after PTX treatments, changes in IL-6 in diabetic rat sera were observed only after GLI or PTX100 treatments, that significantly reduced this cytokine level as related to untreated diabetic rats.

PTX may exert a number of renoprotective effects, besides its role in attenuating nephropathy, by decreasing malondialdehyde levels [51] and simultaneously restoring intracellular glutathione and the oxidative injury to the kidneys. The drug benefits kidneys by stabilizing the renal function and glomerular filtration rate. Concurrent decreases in inflammatory markers, such as TNF-alpha, IL-6 and high-sensitivity C-reactive protein reflect the attenuation of inflammatory damage to the kidneys [18].

By virtue of its antioxidant properties, PTX also mitigates and reduces renal damage in several associated pathologic conditions [52,53]. Diabetes determines oxidative stress in the liver, characterized by increased concentration of ROS and reduction in antioxidant defenses. Such oxidative unbalance in liver cells may play a relevant role in the genesis of diabetic liver disease, as shown recently [54]. PTX may attenuate oxidative stress, by induction of MnSOD and direct scavenging of free radicals and, as a consequence, it inhibits the activities of NF-kB and AP-1 transcription factors [18,55], effects regarded to be crucial in inhibiting pro-inflammatory cytokines. Furthermore, oxidative stress plays a pivotal role in the development of diabetes complications, and overexpression of SOD, in transgenic diabetic mice, prevents diabetic retinopathy, nephropathy and cardiomyopathy [56].

In the present work, we demonstrated that PTX decreased nitrite contents in sera of diabetic rats, as related to those of untreated-diabetic animals. Accordingly, PTX also decreased immunoreactivity for iNOS. This inducible enzyme has been implicated in many human diseases associated with inflammation, and its deficiency was shown to prevent high fat diet-induced insulin resistance in skeletal muscle, but not in the liver. Earlier results [57] suggest that iNOS plays a role in hyperglycemia and contributes to hepatic insulin resistance in ob/ob mice. Other reports indicate that insulin can down-regulate the iNOS pathway *in vivo* [58]. These results provide evidence that increased NO in diabetes is not only a cause, but also an effect of beta-cell destruction, resulting probably from an immunomodulatory activity of insulin. Besides, there is growing evidence that excess generation of ROS, largely due to hyperglycemia, causes oxidative stress, which further exacerbates the development and progression of diabetes and its complications [59].

PTX also decreased the inflammatory response, as evaluated in diabetic rats by the carrageenan-induced acute model of inflammation. This effect was associated with decreases in TNF-alpha and IL-6 levels in paws, as well as by decreases in IL-6 and serum nitrite concentrations. In addition, we also showed that PTX decreases immunoreactivity for iNOS in paw tissues. We showed anti-inflammatory and antioxidant effects of PTX that improved the general conditions of diabetic rats and significantly decreased the burden frequently associated with diabetes.

A recent work [60] demonstrated that GLI, used in the present work for comparison to PTX, decreased intracellular ROS and mitochondrial activity in macrophages. Glibenclamide or glyburide, is an antidiabetic second generation sulfonylurea was shown to present a potent anti-inflammatory effect, as demonstrated by decreases in pro-inflammatory cytokines and nitrite in paws and sera from diabetic rats. Others [61] evidenced the anti-inflammatory effect of GLI in an *ex vivo* model of human endotoxinaemia. Later [62-64], the anti-inflammatory-related effects of GLI in several experimental models *in vivo* were also demonstrated. Interestingly, this anti-inflammatory action was shared by another sulfonylurea, chlorpropamide, in diabetic rats [65].

Previous clinical and experimental studies indicated that PTX can improve cerebrovascular circulation and reduce cerebral edema [66]. PTX mechanism of action includes rheologic effects, as enhanced red cell deformabilitry, alterations in leukocyte activation and modification of coagulation parameters, among others. Later, another work [67] showed that PTX exerts an anti-edematous effect and improves neurological motor dysfunction, in a focal cerebral ischemia model in rats. Furthermore, most likely PTX effects in diabetic rat paw edema is associated, at least partly, with its rheological properties.

Despite the cause for inflammation in diabetes being still under investigation, ROS are a primary candidate. Thus, targeting the cytokine signaling mechanism of oxidative stress/inflammation processes could improve therapeutic options for diabetes and its complications [68]. Interestingly, several of PTX effects as seen in the present study were shared by GLI, a second generation antidiabetic sulfonylurea drug. Thus, in this context, by reducing inflammatory markers and oxidative stress [69,70], as well as by its other multiple effects, PTX may be a useful drug in translation studies and clinical trials for the treatment of diabetes.
